# The Analysis of Surface EMG Signals with the Wavelet-Based Correlation Dimension Method

**DOI:** 10.1155/2014/284308

**Published:** 2014-04-27

**Authors:** Gang Wang, Yanyan Zhang, Jue Wang

**Affiliations:** Key Laboratory of Biomedical Information Engineering of Ministry of Education, Institute of Biomedical Engineering, School of Life Science and Technology, Xi'an Jiaotong University, 28 Xianning West Road, Xi'an 710049, China

## Abstract

Many attempts have been made to effectively improve a prosthetic system controlled by the classification of surface electromyographic (SEMG) signals. Recently, the development of methodologies to extract the effective features still remains a primary challenge. Previous studies have demonstrated that the SEMG signals have nonlinear characteristics. In this study, by combining the nonlinear time series analysis and the time-frequency domain methods, we proposed the wavelet-based correlation dimension method to extract the effective features of SEMG signals. The SEMG signals were firstly analyzed by the wavelet transform and the correlation dimension was calculated to obtain the features of the SEMG signals. Then, these features were used as the input vectors of a Gustafson-Kessel clustering classifier to discriminate four types of forearm movements. Our results showed that there are four separate clusters corresponding to different forearm movements at the third resolution level and the resulting classification accuracy was 100%, when two channels of SEMG signals were used. This indicates that the proposed approach can provide important insight into the nonlinear characteristics and the time-frequency domain features of SEMG signals and is suitable for classifying different types of forearm movements. By comparing with other existing methods, the proposed method exhibited more robustness and higher classification accuracy.

## 1. Introduction

The surface electromyographic (SEMG) signals can be collected noninvasively at the surface of the skin by the appropriate electrodes. Because the SEMG signals are approximately the accumulation of all motor unit action potentials (MUAP) around the pick-up area of the electrodes, they reflect the neuromuscular activities of the examined muscle [1]. In addition, these complex signals hold stochastic nature that depends on anatomical and physiological properties of the contacting muscle [2]. At present, the SEMG signals have been widely applied to muscle fatigue assessment [3], functional electrical stimulation (FES) [4], and clinical diagnosis [5]. On the other hand, much research has been done on the control system for powered prostheses [6–12]. In terms of using prosthetic limbs, the inaccurately identified movements would seriously hurt the amputee and result in a frustrated feeling to the user [12]. The development of methodologies to extract the effective features still remains as a primary challenge for the classification of SEMG signals. Hence, the aim of this paper is to investigate the extraction of effective features from SEMG signals.

Many feature parameters have been successfully applied to the classification of SEMG signals. Hudgins et al. extracted some simple time-domain statistics such as integral of absolute value (IVA), zero crossing (ZC), variance (VAR), Willison amplitude (WAMP), and histogram of EMG (HEMG) from upper limb SEMG signals [12]. Due to the stochastic nature of the raw SEMG signals, Kang et al. extracted the AR coefficients and the cepstral coefficients of SEMG signals by autoregressive models for the signature discrimination [13]. Hu and Nenov employed multivariate AR models to extract the features vectors of SEMG signals as control commands of artificial limbs [14]. Chen et al. proposed the discriminant bispectrum feature to distinguish a set of hand and wrist motions [11]. In the frequency domain analysis, Peleg et al. identified different finger activation by using the Fourier transform coefficients [15]. In addition, time-frequency transforms have been also paid considerable attention in the field of SEMG signal processing. Englehart et al. extracted some features based on the short-time Fourier transform, the wavelet transform, and the wavelet packet transform to improve the accuracy of transient SEMG signals classification [1, 16]. Wang et al. presented the optimal wavelet packet method based on Davies-Bouldin criterion to discriminate four types of prosthetic movements [8]. Recently, Pinzon-Morales et al. successfully recognized five hand movements by means of combining Hilbert-Huang analysis with a fuzzy clustering classifier [17]. Due to the nonlinear nature of SEMG signals, Arjunan and Kumar used the fractal dimension and the maximum fractal length as fractal features to decode four categories of wrist and finger flexions [18]. However, these methods only characterized the SEMG signals by single analysis method and cannot adequately represent the SEMG signals for prosthetic pattern recognition. It is essential to combine the different analysis methods to further extract the effective features of SEMG signals because the enhanced classification accuracy can benefit the usage of powered prosthetic limbs controlled by the SEMG signals. On one hand, previous studies have demonstrated that the SEMG signals have nonlinear characteristics [19, 20]. In addition, from the point of view of physiological properties, the SEMG signals reflect the electrical activity of the nonlinear neuromuscular system [21]. These studies suggest that, to some degree, nonlinear time series analysis can provide a potentially important insight into the classification of SEMG signals. As is well known, the correlation dimension is one of the commonly used nonlinear analysis methods. In this study, the correlation dimension method was used to describe the nonlinear characterization of the SEMG signals. On the other hand, the wavelet transform method has achieved great success in many applications. Unlike traditional signal processing methods, the wavelet transform can provide many important signal characteristics which are useful to classification. In this study, we combined the nonlinear time series analysis and the time-frequency domain methods to present a wavelet-based correlation dimension approach for the classification of SEMG signals in a prosthetic control system.

## 2. Materials and Methods

### 2.1. Subjects and Data Acquisition

Two channels of SEMG signals were collected by two pairs of bipolar Ag/AgCl electrodes with conductive paste. One pair was located over the flexor carpi radialis (FCR) and the other over the extensor carpi radialis longus (ECRL). All disc electrodes were put on the skin surface of the right forearm of each subject. Electrodes in each bipolar pair had a center-to-center spacing of 20 mm. The reference electrode was an Ag/AgCl disc electrode and was placed on the top side of the wrist. To help minimize the cross interference between the two channels, electrodes of 5 mm diameter were used. Differential amplifiers with bandpass filters between 10 Hz and 500 Hz were used to reduce the effects of high-frequency noises and low-frequency movement artifacts. The sampling frequency was 1000 Hz. The sampling was triggered when the energy value of the SEMG signals based on the myoelectric activity exceeded a set threshold value.

Five normally limbed subjects (three males and two females) took part in this experiment. The mean age of all subjects was 23.2 (±1.64) years. Each subject was instructed to perform four different types of movements: forearm pronation (FP), forearm supination (FS), hand close (HC), and hand open (HO). The subjects were first asked to initiate a movement while the energy value of the SEMG signals was examined with a moving window. After a threshold was exceeded and the sampling was triggered, continuous SEMG samples were recorded for 800 ms with the intention of signals analysis. For each subject, all forearm movements were performed in four trails. One category of forearm movements was repeated for 8 times in each trial. So there were 32 groups of two channels of SEMG signals for each subject and 160 groups in total. In order to avoid mental and muscle fatigue, the subjects were asked to relax for 5 s between each movement and for 1 min between each trial. For comparison, one hand amputee was also recruited in the study. The procedures of data acquisition for hand amputee were the same as the normally limbed subjects.

### 2.2. Methods

The wavelet transform is a local time-frequency analysis method which has the fixed analysis window and the variable resolution both in time domain and frequency domain. Due to the properties of the flexible and self-adaptive multiresolution of the wavelet function, the wavelet analysis has been widely applied in very diverse problems [22–24]. In this study, the analysis of SEMG signals was performed using a discrete wavelet transform (DWT) with adequate scale values. This approach can translate the signals in the time domain in order to involve the entire SEMG signals. The computation method of the wavelet analysis of the SEMG signals was presented below.

Given that a SEMG signal  *x*(*n*)  is a finite energy signal and is decomposed to level  *J*. Firstly, the original signal  *x*(*n*)  was filtered through a discrete low-pass filter  *h*_0_  and a high-pass filter  *h*_1_, which resulted in an approximation signal and a detail signal. The approximation signal describes the high-scale and low-frequency components of the signal and the detail signal describes the low-scale and high-frequency components of the signal. Secondly, the approximation signal was further decomposed into a second-level approximation signal and detail signal. Finally, this process was repeated until level  *J*  was achieved. The original SEMG signal  *x*(*n*)  can be denoted by the approximation signal  *A*_0_  at level 0. Then, the approximation signal  *A*_*j*_  and the detail signal  *D*_*j*_  of the DWT at level  *j*  can be expressed by

(1) $$\begin{array}{c} A_{j}\left(n\right)=\underset{k\in Z}{\sum }h_{0}\left(k-2n\right)A_{j-1}\left(k\right), \end{array}$$

(2) $$\begin{array}{c} D_{j}\left(n\right)=\underset{k\in Z}{\sum }h_{1}\left(k-2n\right)A_{j-1}\left(k\right), \end{array}$$

where  *k* is the translation parameter in time domain and  *j*  is the scale parameter which determines the inflation or deflation of the wavelet function. The low-pass filter  *h*_0_  is related to the scaling function  *ϕ*  and the high-pass filter  *h*_1_  is related to the wavelet function  *ψ*. These functions can be defined as

(3) $$\begin{array}{c} \phi_{j,k}\left(n\right)=2^{-j/2}\phi \left(2^{-j}n-k\right)\\ \psi_{j,k}\left(n\right)=2^{-j/2}\psi \left(2^{-j}n-k\right), \end{array}$$

which has the same parameters as (1) and (2). The detail signal in (2) is also called the wavelet coefficients, which can be represented by  **d**_*j*_ = {*d*_*j*_(*k*) | *k* = 1,2,…, *K*_*j*_}. If the depth of wavelet analysis is too small, the classification information would not be extracted efficiently. If the depth is too large, the computation complexity would be increased remarkably. Therefore, in this study, the SEMG signals were decomposed to the fourth level using the DWT. The Coiflet wavelet of order 5 was selected as the mother wavelet, because the waveform of Coiflet wavelet can fit the SEMG signals appropriately.

The physiological signals generated by complex self-regulating systems may exhibit complex temporal fractal patterns in which the large scale structure of the signal resembles the small scale structures, as in the branchings of a tree. The fractals can exhibit this self-similar property and the fractal dimension can be used to characterize the fractal properties of a structure. Hence, the fractal dimension has been widely applied in the analysis of the physiological signals which has self-similar property [18, 25, 26]. The most common fractal dimensions include Hausdorff dimension, similar dimension, Kolmogorov capacity dimension, box dimension, information dimension, and correlation dimension. In this study, the correlation dimension was used to measure the fractal property of the SEMG signals and extract the features of the SEMG signals in order to classify different movements.

The correlation dimension can be directly calculated from the time series by using the GP algorithm proposed by Grassberger and Procaccia [27, 28]. This algorithm is based on the embedding theorem and the phase space construction and has become the most widely used approach for estimating the fractal dimensions of experimental data sets. Let  *D* = {*d*_*k*_, *k* = 1,…, *K*}  denote the wavelet coefficients corresponding to a fixed resolution level of the multiresolution analysis. Then, a new phase space can be reconstructed by embedding the time series  *D*  into an *m*-dimensional Euclidean space and the elements of this new phase space can be given by

(4) $$\begin{array}{c} D_{n}\left(m,\tau \right)=\left\{d_{n},d_{n+\tau },\ldots ,d_{n+(m-1)\tau }\right\},\;\left(n=1,2,\ldots ,N\right), \end{array}$$

where  *m*  is the embedding dimension,  *τ*  is the delay time, and  *N*  is the number of points in the reconstructed phase space (*N* = *K* − (*m* − 1)*τ*). For a reference point  *D*_*i*_  selected from  *N*  points of the reconstructed phase space, the distance between the reference point and the other  *N* − 1  points can be defined as

(5) $$\begin{array}{c} r_{ij}=\text{dis}\left(D_{i},D_{j}\right)={\left[\overset{m-1}{\underset{k=0}{\sum }}{\left(d_{i+k\times \tau }-d_{j+k\times \tau }\right)}^{2}\right]}^{1/2},\;\left(i\neq j\right). \end{array}$$

By repeating the calculation procedure of the distance described above, for all  *D*_*i*_  (*i* = 1,…, *N*), the correlation integral function can be obtained by

(6) $$\begin{array}{c} C_{m}\left(r\right)=\frac{1}{N\left(N-1\right)}\overset{N}{\underset{i=1}{\sum }}\overset{N}{\underset{j=1}{\sum }}\theta \left(r-r_{ij}\right),\;\left(i\neq j\right), \end{array}$$

where the correlation integral is defined as the probability in which the points are included around the reference point according to the radius of  *r*  in the *m*-dimensional phase space and the Heaviside function  *θ*  is defined as

(7) $$\begin{array}{c} \theta \left(x\right)=\{\begin{array}{cc} 1 & \text{if}\;\;x>0,\\ 0 & \text{if}\;\;x\leq 0. \end{array} \end{array}$$

Because the parameter  *N*  is usually far greater than 1, (6) can be approximately represented by

(8) $$\begin{array}{c} C_{m}\left(r\right)=\frac{1}{N^{2}}\overset{N}{\underset{i=1}{\sum }}\overset{N}{\underset{j=1}{\sum }}\theta \left(r-r_{ij}\right),\;\left(i\neq j\right). \end{array}$$

When the parameter  *r*  is sufficiently small, the correlation integral function can be given by

(9) $$\begin{array}{c} \ln C_{m}\left(r\right)=\ln C+D_{c}\ln r. \end{array}$$

Consequently, the correlation dimension  *D*_*c*_  can be estimated by

(10) $$\begin{array}{c} D_{c}=\underset{r\to 0}{\lim}\left(\frac{\partial \ln C_{m}\left(r\right)}{\partial \ln r}\right). \end{array}$$

In practice, the correlation dimension  *D*_*c*_  can be calculated from the slope of the straight line of best fit in the linear scaling range region of a plot of  ln⁡*C*_*m*_(*r*)  versus  ln⁡*r*  [28]. In this study, because continuous SEMG signals of 800 ms were recorded and the sampling rate was 1000 Hz, the number of wavelet coefficients  *K*  was equal to 800 in the multiresolution analysis. In addition, it is very important to choose appropriate values of the embedding dimension  *m*  and the delay time  *τ*  in the reconstructed phase space as their precision is associated with the accuracy degree of the invariants characterizing the strange attractors. A large number of investigations demonstrated that the embedding dimension and the delay time were correlated with each other since the time series could not have infinite length and could be inevitably contaminated by various noises in the real environment. The C-C algorithm, which was developed by Kim et al. [29], was a combination approach for estimating optimum embedding dimension and delay time. Firstly, the delay time  *τ*  can be calculated by using the correlation integral and the BDS statistic. Secondly, a suitable value can be achieved for the delay time window  *τ*_*w*_  which is the entire time spanned by the components of each embedded point. Finally, the embedding dimension  *m*  can be obtained by the formula  *τ*_*w*_ = (*m* − 1)*τ*. By using the C-C method in this study, the embedding dimension  *m*  and the delay time  *τ*  were determined as 16 and 10, respectively. After identifying  *m*  and  *τ*, the number of points in the reconstructed phase space could be calculated as 650.

## 3. Results

The SEMG signals were firstly segmented from the onset of a contraction by the length of 800 samples. The amplitudes of segmented SEMG signals were normalized to avoid the effect of different amplitude scales on the following calculation. Specifically, the segmented SEMG signals were normalized to center it at zero mean and scale it to unit standard deviation. For each subject, the SEMG signals were then analyzed by the wavelet transform. The SEMG signals are a result of the summation of the similar MUAPs originated from different locations of the muscles. Because the patterns of SEMG signals obtained at one sampling rate are statistically similar to patterns obtained at other sampling rates, the SEMG signals have the property of self-similarity [19]. Therefore, after decomposing the SEMG signals by the wavelet transform, the correlation dimension was calculated to extract the features of the SEMG signals. In the end, the wavelet decomposition and the computation of correlation dimension were performed on each channel individually and the features of both channels were combined to obtain the new feature set in order to classify four different classes of movements.

**Figure 1:**
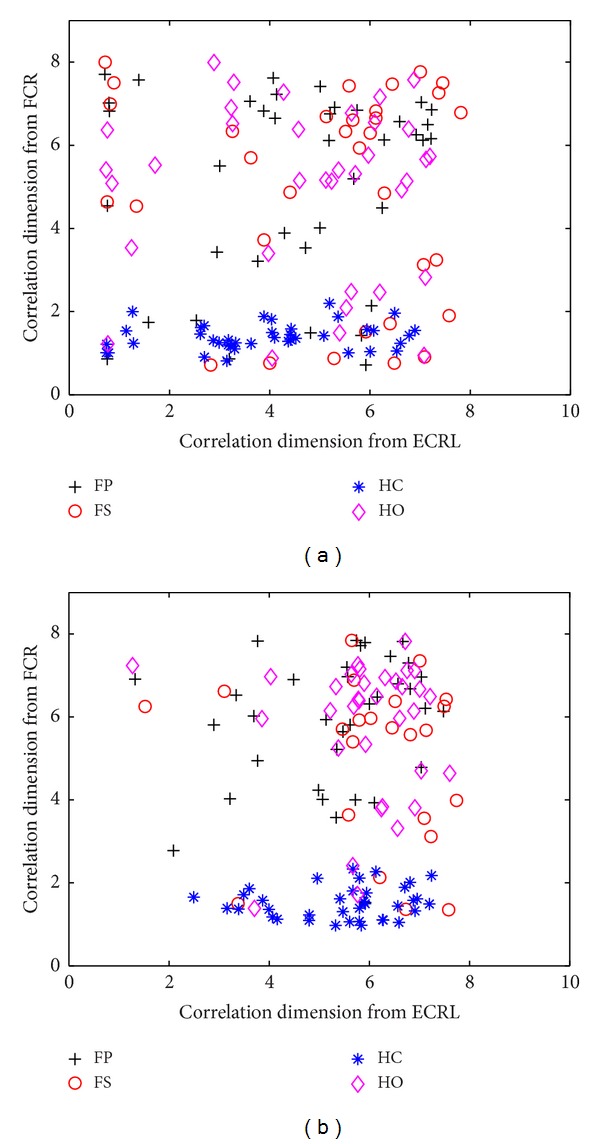
Scatterplot of the wavelet-based correlation dimension for two channels of SEMG signals recorded from the FCR and the ECRL in five normally limbed subjects at the first resolution level (a) and the second resolution level (b).

In this study, the classification of SEMG signals was performed for five normally limbed subjects and one hand amputee, respectively. At the first and the second resolution level, the classification results of the wavelet-based correlation dimension method for two channels of SEMG signals recorded from the flexor carpi radialis (FCR) and the extensor carpi radialis longus (ECRL) in five normally limbed subjects are summarized in Figure 1. From the scatterplot of Figure 1(a), it can be observed that the scatterplot of the correlation dimension of four movements overlaps heavily at the first resolution level, and we cannot distinguish four different classes of movements. At the second resolution level, the scatterplot of the correlation dimension of four movements tends to be more concentrated than those of the first resolution level and four different types of movements are still not able to be distinguished from each other.

**Figure 2:**
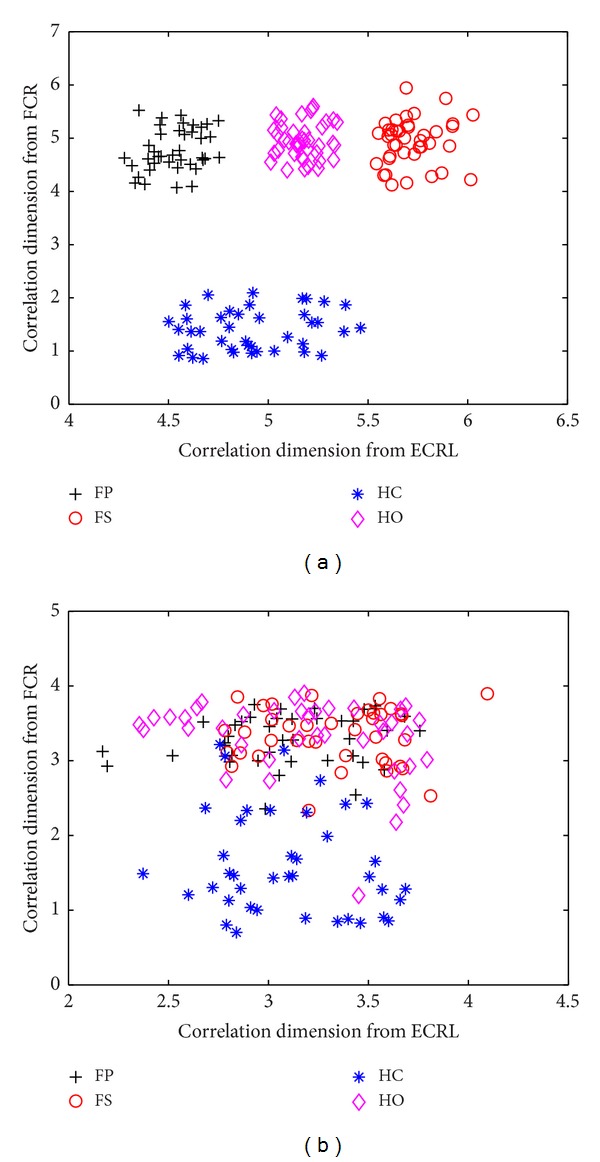
Scatterplot of the wavelet-based correlation dimension for two channels of SEMG signals recorded from the FCR and the ECRL during four different classes of forearm movements in five normally limbed subjects at the third resolution level (a) and fourth resolution level (b).

For the third and the fourth resolution level, the scatterplots of the wavelet-based correlation dimension of two channels of SEMG signals in five normally limbed subjects are illustrated in Figure 2. From Figure 2(a), it can be observed that there are four separate clusters corresponding to different forearm movements at the third resolution level and the cluster relevant to HC movements was away from the other three movements. The values of range and standard deviation of the wavelet-based correlation dimension for SEMG signals recorded from the FCR and the ECRL can be observed in Table 1. The Gustafson-Kessel (GK) algorithm is a powerful clustering technique and has been widely applied to various domains including image processing and pattern recognition and classification [30–32]. This approach is an extension of the fuzzy c-means algorithm and seeks to minimize the fuzzy sum of squared generalized distances of the data to the cluster centroids by employing an adaptive distance norm and estimating the distance-inducing covariance matrix. Thus, the GK algorithm allows each cluster to locally adapt the distance metric to different geometrical shapes of the data. Since the shape of the clusters presented in Figure 2(a) resembled ellipses, the GK clustering algorithm was used to classify the SEMG signals. In this study, the number of clusters was set at 4, the weighting exponent was 2, the maximum number of iterations was assigned to 10000, and the convergence tolerance was 0.00001. The resulting classification accuracy was 100% in Figure 2(a). From Figure 2(b), it can be indicated that the scatterplot of the wavelet-based correlation dimension of four movements also overlaps together for the fourth resolution level and four different types of movements cannot be distinguished from each other. Additionally, for one hand amputee, when the SEMG signals were decomposed at the third resolution level by using the wavelet transform, the classification accuracy also achieved 100% by means of the GK clustering classifier.

## 4. Discussion

In this study, we have examined the ability of classifying four different types of forearm movements by using the SEMG signals from five normally limbed subjects and one hand amputee. All subjects were asked to perform four different types of movements: forearm pronation (FP), forearm supination (FS), hand close (HC), and hand open (HO). The SEMG signals of these subjects were acquired and were decomposed by the wavelet analysis. Then, the wavelet-based correlation dimension of the SEMG signals was calculated to extract the features of different movements by using the GP algorithm [27, 28]. Because the different movements result in the different activities of the nervous system and the wavelet-based correlation dimension can catch these different nervous activities, the wavelet-based correlation dimension method can represent the difference of the SEMG signals relevant to different movements. Our results validated the possibility of improving the classification accuracy of SEMG signals by using the wavelet-based correlation dimension method.

**Figure 3:**
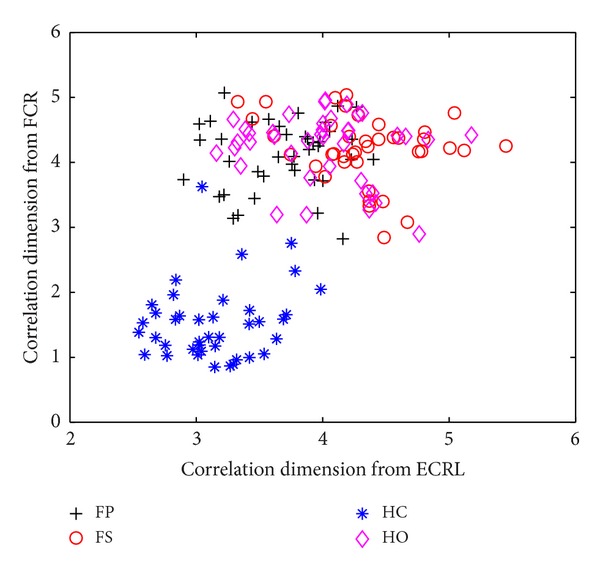
Scatterplot of the correlation dimension for two channels of SEMG signals recorded from the FCR and the ECRL during four different classes of forearm movements.

In order to evaluate the performance of the wavelet-based correlation dimension method, the comparison with other algorithms was made. We used the correlation dimension without multiresolution analysis in this comparison task. To facilitate the effectiveness of the comparison, this method was applied to the same database of the SEMG signals. The length of the analysis window of SEMG signals was also selected as 800 ms. The calculation of the correlation dimension was directly performed on the SEMG signals without the multiresolution analysis. The scatterplots of the correlation dimension without multiresolution analysis for five normally limbed subjects are illustrated in Figure 3. Comparing with the wavelet-based correlation dimension method, the direct correlation dimension method was completely invalid in terms of distinguishing four different classes of movements by using the same SEMG signals. This result was possibly caused by the difference of two feature extraction methods. The SEMG signals used in this study were recorded by differential amplifiers with bandpass filters between 10 and 500 Hz. Since no further preprocessing was implemented, our SEMG signals might contain noise and interference. As for the wavelet-based correlation dimension method, the polynomial trends of the signals are automatically removed from the original signals during the procedure of wavelet transformation because of the vanishing moment possessed by the wavelets [33]. To some extent, this procedure will reduce the influence of noise on our data analysis. This reveals that the wavelet-based correlation dimension method has more robust capability than the direct correlation dimension method for identifying four different classes of forearm movements when noise exists.

In addition to the correlation dimension method, we compared the wavelet-based correlation dimension method with the wavelet packet transform (WPT) method which was demonstrated to offer the best performance for the classification of SEMG signals [1]. A coif5 wavelet was also chosen as the mother wavelet and the GK clustering classifier was used to classify the SEMG signals in this study. On the other hand, the time-domain (TD) method was also used in this comparison task. The integral of absolute value, the zero crossing, the variance, and the waveform length were employed to represent the TD features in this study.  Table 2 exhibits the classification results of three methods for each normally limbed subject. It could be observed from this table that the mean classification accuracy using the wavelet-based correlation dimension method was higher than that of the WPT method by 8.125%. Because the wavelet-based correlation dimension method combined the nonlinear time series analysis and the time-frequency domain methods, the proposed method can provide more useful information for the classification of SEMG signals than the WPT method. Therefore, the wavelet-based correlation dimension method is more effective than the WPT method when extracting the features of the SEMG signals. Additionally, the mean classification accuracy of the WPT approach was higher than that of the TD approach by 6.875%. As is well known, the WPT method is a joint time-frequency analysis approach, which demonstrates that the WPT method can represent the characteristics of SEMG signals better than the TD method. This results in high classification accuracy for the WPT approach.

**Figure 4:**
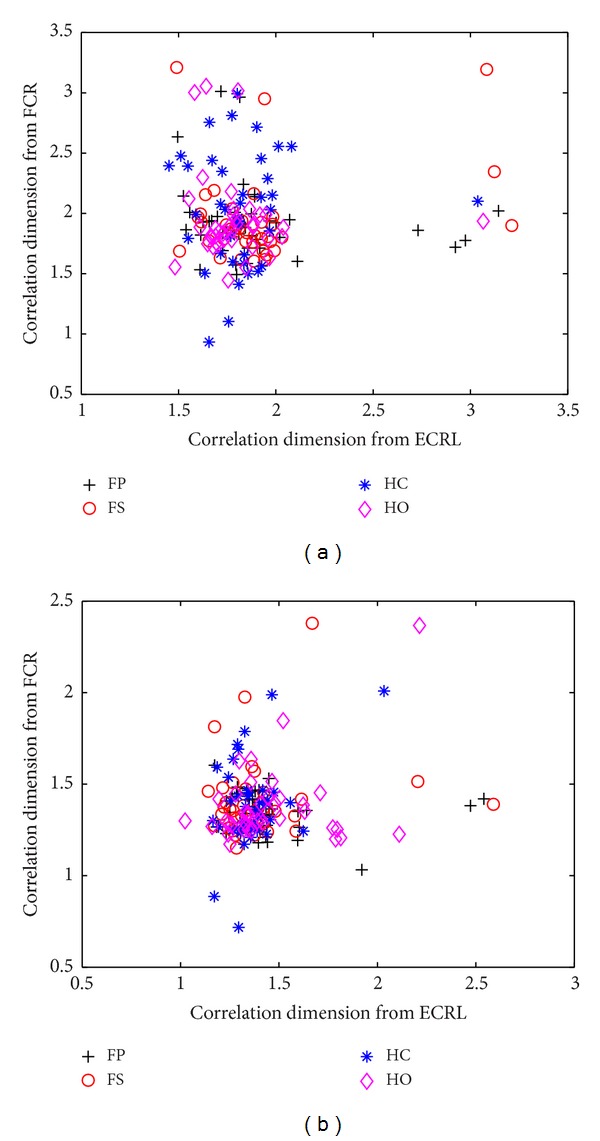
Scatterplot of the wavelet-based correlation dimension for two channels of SEMG signals recorded from the FCR and the ECRL in five normally limbed subjects at the fifth resolution level (a) and the sixth resolution level (b).

For further investigating the wavelet-based correlation dimension method, the SEMG signals were decomposed to the fifth level and the sixth level by using wavelet transform. The correlation dimension was then calculated for the decomposed wavelet coefficients. The classification results of the wavelet-based correlation dimension method for the two-channel SEMG signals recorded from the FCR and the ECRL in five normally limbed subjects are illustrated in Figure 4. From this figure, it can be observed that the wavelet-based correlation dimensions of four movements mix together whether at the fifth resolution level or the sixth resolution level. Furthermore, four different types of movements are difficult to distinguish from each other. In this study, we calculated 6 levels of the correlation dimensions for the SEMG signals during four different movements. The largest separation among four movements is obtained at the third resolution level. This indicates that the decomposed signals can provide the most useful classification information when the SEMG signals are decomposed to the third level by the wavelet transform. For the other levels of the wavelet transform, the classification accuracy would decrease because the noisy information of classification is enhanced or the useful classification information cannot be extracted efficiently.

For the classification of SEMG signals in a prosthetic control system, previous studies focused on the time-domain analysis, the frequency domain analysis, and the time-frequency domain analysis in order to extract the features of the SEMG signals for distinguishing the different forearm movements. These methods had certain limitations because they did not use the nonlinear intrinsic characteristic of the SEMG signals. In this study, according to the nonlinear of the SEMG signals, we combined the time-frequency domain analysis approach and nonlinear dynamics analysis to explore the features of the SEMG signals. Comparing with the traditional SEMG analysis methods, the proposed method in this study can obtain more useful information and can better identify different forearm movements. The experimental results demonstrated that the proposed method can exhibit good performance for the classification of the SEMG signals.

One limitation of this study was that the SEMG database for classification performance evaluation of the SEMG signals was the same as the one used for developing the algorithm and the classification criterion. Therefore, a larger database would be required for assessing the classification performance of the SEMG signals during different forearm movements for this proposed method.

## 5. Conclusions

In summary, the wavelet-based correlation dimension method can provide a novel description of the SEMG signals during the different forearm movements and the SEMG signals can be characterized efficiently by this method. The fractal analysis was performed by the GP algorithm after the SEMG signals were decomposed by the wavelet transform in order to obtain the features of the SEMG signals. Our results reveal that the wavelet-based correlation dimensions of SEMG signals are determined by the different types of forearm movements. Thus, the wavelet-based correlation dimension extracted from two channels of SEMG signals can be employed to distinguish four different types of upper arm movements. By comparing with the correlation dimension method without multiresolution analysis, the wavelet-based correlation dimension method proposed in this study represented more robustness and higher classification accuracy. Although the data analysis approach in this study was applied to the classification of SEMG signals in a prosthetic control system, the wavelet-based correlation dimension method presented here provides a new idea for analyzing other nonstationary physiological signals and can be easily generalized to other applications.

## Figures and Tables

**Table 1:** The range and standard deviation of the wavelet-based correlation dimension of SEMG signals recorded from the FCR and the ECRL for different forearm movements.

Forearm movement	FCR			ECRL		
	Min value	Max value	Standard deviation	Min value	Max value	Standard deviation
Forearm pronation	4.071	5.524	0.399	4.279	4.754	0.128
Forearm supination	4.122	5.945	0.429	5.543	6.027	0.1269
Hand close	0.859	2.1	0.378	4.501	5.463	0.268
Hand open	4.403	5.596	0.335	5.012	5.346	0.094

**Table 2:** Classification results of three methods: the wavelet-based correlation dimension method, the WPT method, and the TD method.

Classification accuracy (%)	Subject					Mean
	1	2	3	4	5	
Correlation dimension	100	100	100	100	100	100
WPT method	93.75	90.625	87.5	96.875	90.625	91.875
TD method	87.5	81.25	81.25	90.625	84.375	85
